# Advances in non-small cell lung cancer mechanomedicine: deciphering the signaling networks that govern tumor-TME interactions

**DOI:** 10.1186/s13046-024-03242-1

**Published:** 2024-11-30

**Authors:** Antonios N. Gargalionis, Kostas A. Papavassiliou, Efthimia K. Basdra, Athanasios G. Papavassiliou

**Affiliations:** 1 Laboratory of Clinical Biochemistry, Medical School, ‘Attikon’ University General Hospital, National and Kapodistrian University of Athens, 12462 Athens, Greece; 2 First University Department of Respiratory Medicine, Medical School, ‘Sotiria’ Chest Hospital, National and Kapodistrian University of Athens, 11527 Athens, Greece; 3 Department of Biological Chemistry, Medical School, National and Kapodistrian University of Athens, 11527 Athens, Greece

**Keywords:** Non-small cell lung cancer, Tumor microenvironment, Mechanomedicine, Mechanosignaling, Mechanotransduction, YAP, TAZ

## Abstract

Cells from the tumor microenvironment (TME) interact with tumor cells in non-small cell lung cancer (NSCLC) to form a reciprocal crosstalk which influences tumor growth, proliferation, metastasis and multidrug response. This crosstalk is modulated by TME mechanical inputs, which elicit the processes of mechanosensing and mechanotransduction. Recent advances in unveiling these signaling networks establish the interdisciplinary field of mechanomedicine to exploit emerging diagnostic, predictive and therapeutic tools for more effective NSCLC treatments.

The cell and extracellular matrix (ECM) composition shape a distinct tumor microenvironment (TME) in non-small cell lung cancer (NSCLC), which regulates the properties of cancer cells and, importantly, response to applied therapy. TME with its cell components, including cancer-associated fibroblasts (CAFs), endothelial cells and immune cells, provides physical support for cancer cells and mechanisms of immune evasion against the anti-tumor immune response. Apart from biochemical signals, such as alterations in pH, oxygen abundance, immune surveillance and the inflammatory status, mechanical cues represent a crucial factor that controls TME composition itself and, ultimately, cell survival, invasion and drug response [1]. Solid stress, shear stress, alterations of ECM rigidity and changes in the topology of TME cellular components are mechanical inputs which evoke corresponding responses of TME and cancer cells. These responses define the processes of mechanosensing and mechanotransduction during tumor development and progression. Protein mechanosensors comprise integrins, focal adhesion proteins, mechano-stimulated ion channels, proteins of the cytoskeleton and mechano-induced transcription factors such as yes-associated protein (YAP) and transcriptional coactivator with PDZ-binding motif (TAZ) [2]. Mechanosignaling affects responses to chemotherapy by triggering processes of cancer cells which are associated with resistance to chemotherapy, like epithelial-to-mesenchymal (EMT) transition and cancer cell stemness [3]. Therefore, this dynamic interplay between mechanical inputs, TME and NSCLC cells provides potential new intervention strategies in the clinical setting.

Among different physical cues, alterations of the ECM stiffness have been implicated in treatment efficiency. ECM stiffness plays a critical role in cancer initiation and progression by regulating aggressive phenotypes of cancer cells. Activation of CAFs, increased collagen crosslinking and enhanced accumulation of ECM components are key factors that drive ECM stiffness [4]. Chemotherapy, targeted therapy and immunotherapy mechanisms of treatment crosstalk with mechanotransduction pathways to determine drug efficacy [3]. Latest data reveal that ECM forms a physical barrier that particularly inhibits infiltration by immune cells, hinders efficient delivery of immunotherapy, therefore different strategies targeting ECM have been proposed to improve sensitivity to immunotherapy [4]. These aberrations of mechanotransduction networks provide a rationale for designing new combinatorial treatments to overcome resistance to treatment regimens.

In NSCLC, ECM collagen activates resistance of NSCLC cells to epidermal growth factor receptor (EGFR)-tyrosine kinase inhibitors (TKIs) through the integrin-*β*1 mechanoreceptor in a dose-dependent manner. This dose-dependent effect could be a matter of clinical relevance to overcome drug resistance [5]. Regarding the role that TME cells play in these signaling networks, it has been established that CAFs produce molecules, such as transforming growth factor beta (TGF-*β*), and can reshape the ECM, alter ECM rigidity and suppress the anti-tumor immune response [6]. Wu et al. suggest that CD248 (endosialin) is a specific biomarker for NSCLC-derived CAFs, which promotes metastasis by triggering ECM stiffness via activation of the Hippo signaling pathway. CD248-expressing CAFs mediate ECM stiffness and foster infiltration and migration of NSCLC cells in vitro and in vivo. Mechanistically, the upregulation of the Hippo pathway induces the expression of connective tissue growth factor (CTGF), subsequent regulation of collagen I homeostasis and modulation of ECM rigidity [7]. Accordingly, targeting ECM stiffness through TGF-*β* blockade is under clinical testing in NSCLC with the monoclonal antibody fresolimumab (NCT02581787) [2]. There are also promising findings showing that patients with metastatic breast cancer who received high dose of fresolimumab demonstrated higher median overall survival than those who received the low dose, accompanied with augmented tumor-specific CD8 levels [8]. Furthermore, ECM stiffness regulates the mechano-effector YAP and the expression of programmed death-ligand 1 (PD-L1) in lung adenocarcinoma, while YAP affects PD-L1 expression under stiffer matrices [9]. YAP has been identified as a potential effector of the TME − NSCLC cells interaction that facilitates cancer growth. YAP enhances the expression of PD-L1 through binding of the YAP/TEA domain family member (TEAD) transcriptional complex to the *PD-L1* promoter in EGFR-TKI-resistant lung adenocarcinoma. Consequently, targeting YAP could alleviate NSCLC TME from distorted ECM stiffness and concomitant effects on PD-L1 expression [10, 11].

TME drives adaptive mechanisms of drug resistance through CAFs and other types of TME cells including immune cells and cells of the vasculature system. Immune cells secrete cytokines and chemokines which inhibit targeting of tumor cells by cytotoxic T cells and are also implicated in chemotherapy outcomes [12]. Aside from CAFs, alternative TME types of cells appear as intermediates between regulation of ECM rigidity, NSCLC properties and levels of treatment responses. Tripartite motif-containing 28 (TRIM28; also known as transcriptional intermediary factor 1β) induces potentiated nuclear factor-kappa beta (NF-κB) signaling by physically interacting with receptor-interacting serine/threonine-protein kinase 1 (RIPK1). NF-κB potentiation triggers recruitment of the TME myeloid-derived suppressor cells (MDSCs) and abrogates infiltration of activated CD8^+^ T cells, thus leading to anti-PD-1 treatment resistance because of immune evasion. Silencing of TRIM28 can restructure the TME and restore anti-programmed death protein-1 (PD-1) treatment efficacy, rendering TRIM28 a promising TME-associated pharmacological target for inhibition [13]. Additionally, a distinct subset of CD4^+^PD-1^+^ chemokine (C-X-C motif) ligand 13 (CXCL13)^+^ T cells has been recently shown to physically interact with antigen-presenting cells in the TME of NSCLC. This distinct subset of T-helper tumor (Tht) cells is essential for anti-PD-1 efficient treatment [14]. Tumor-associated macrophages (TAMs) represent an additional type of TME cells, and their polarization is known to modulate tumor immune responses. ECM stiffness and TAMs polarization influence immune exclusion, which decreases the efficiency of immunotherapy [15]. Given the pivotal role that TME plays in NSCLC progression, a novel TME eight-gene signature associated with the tumor mutational burden and overall survival has been revealed, which potentially forms a new prognostic tool for lung adenocarcinoma [16]. Considering these findings, accumulating data indicate that it is critical to decode mechanosignaling between TME and NSCLC cells under different ECM conditions towards finding novel combinatorial treatments and strategies, prognostic and predictive tools, but also guide ECM targeting more efficiently.

Protein effectors of mechanotransduction have been suggested to play important roles in cancer initiation, progression, metastasis and treatment responses. YAP transcriptional regulator mediates resistance to RAF and mitogen-activated protein kinase (MAPK) kinase (MEK)-targeted cancer therapies [17], YAP and TAZ mediate resistance of melanoma cells to BRAF inhibitors [18], whereas recent findings reveal that Piezo1 mechano-induced ion channel regulates myeloid cells to control innate immunity against cancer [19]. The aberrant function of mechanotransduction effector proteins also seems to ease mechanisms of drug resistance in NSCLC [20]. Elevated TAZ expression is correlated with poor overall survival of NSCLC patients, while YAP and TAZ seem to have an oncogenic function in NSCLC, thereby representing independent prognostic factors of the disease and potential therapeutic targets [21]. Moreover, pharmacological inhibition of Piezo ion channels, a typical set of excitatory ion channels directly gated by mechanical forces, is in its first steps [3]. G protein-coupled receptors (GPCRs) are also a mechanosensitive family of receptors that is prominently targeted in preclinical and clinical settings. Pepducin inhibitors (small-lipidated peptides created from the intracellular loops of GPCRs) can target protease-activated receptor 1 (PAR1), a member of the PAR family of GPCRs. PAR1 inhibition displays decreased migration in primary lung cancer cells and lung cancer cell lines and disrupts PAR1/extracellular signal-regulated kinase (ERK) 1/2 pathway [22]. Given these observations, targeting of mechanosensory receptors and signaling molecules should be further incorporated into the design of preclinical and clinical testing.

Alternative approaches tend to exploit mechanobiological properties to achieve better therapeutic outcomes. Experiments from NSCLC cells and lung fibroblasts subjected to mechanical stress show that mechanical load regulates cell rearrangement, cytoskeletal remodeling and long-term stretching can alter the alignment of cells and the length of mitochondria [23, 24]. Lung cancer cells under mechanical load that simulates lung expansion and contraction exhibit changes in morphology, decrease of growth rate, reduction of proliferation and diminish the efficiency of chemotherapeutic drugs [25]. Taken together, mechanobiology-based therapies are emerging regimens to target cancer cells through mechanical dysregulations. These therapies incorporate mechanical stretch, shock wave, high intensity ultrasound frequency and low intensity pulsed ultrasound [2]. Application of novel folate-nanobubbles in combination with therapeutic ultrasound has been shown to eliminate tumor cells in vitro, suppress tumor growth and increase overall survival in vivo [26]. Recent data also reveal that cyclic stretching causes apoptosis in tumor cells on soft substrates through regulation of the Piezo1 calcium channel [27].

Although the aforementioned mechano-based therapies seem promising, further clarification of the molecular mechanisms through which they selectively eliminate cancer cells is necessary. Different types of solid tumors demonstrate varying degrees of ECM stiffness, therefore the mechanotransduction properties in each clinical setting must be fully elucidated to achieve efficient clinical application. In corroboration, although there were promising findings in preclinical evaluation of targeting mechanosensory molecules, clinical trials assessing antibodies against mechanotransduction proteins failed. Modulation of mechanical properties via shock wave, mechanical stretch, high intensity ultrasound frequency and low intensity pulsed ultrasound also pose risks of nonselective killing of cancer cells along with normal cells [2].

One of the major obstacles to embody aspects of mechanobiology and TME in the clinic is the validation of technological platforms and standardized methods. There must be an international consensus of methodological approaches to reach reliable application of potential prognostic, predictive and diagnostic markers involving novel technology platforms for cancer cell mechanobiology studies. Mechanobiology research is a multiscale process which incorporates computational and imaging techniques. Mechanical stimulation is evaluated in multilevel experimental settings which involve animal models at the organ level, tissue engineering bioreactors at the tissue level, cell culture models at the cellular level and molecular analysis at the molecular level. Several assays and imaging tools have been also developed to measure the biochemical and structural responses to this mechanical stimulation [28]. To this end, methods have been suggested to provide quantification of cellular mechanical forces [29]. Traction force microscopy (TFM) has been proposed to measure forces generated by single cells. TFM has been widely established as a method for measuring these forces and this relies on the fact that TFM produces quantified force maps by calculating the strains which are developed at cell adhesions in conjunction with soft substrates. The technique has been further developed to model the 3-dimensional (3D) cellular forces exerted on planar 2D surfaces. Nevertheless, there are issues raised regarding the setup of experimental procedures and data analysis which still limit its use in research laboratories [30]. Additional methods include modulation of intracellular forces through molecular targeting, manipulation of ECM stiffness though alterations in the degree of crosslinking of polymeric hydrogels, application of local force at single adhesion sites by using magnetic tweezers (MTs), optical tweezers (OTs) and atomic force microscopy (AFM) [29].

Recent methods, such as molecular imaging and high-throughput multiplex immunohistochemical imaging of the TME have been developed to achieve the identification of different TME and ECM components. Validation and advancement of these methods could constitute a non-invasive approach of NSCLC molecular classification [31]. In addition, a histology-based computational staining has been developed to cartograph the TME in lung cancer. Certain patterns have been correlated with expression profiles of signaling pathways and with patient survival [32].

## Conclusion

**Fig. 1:**
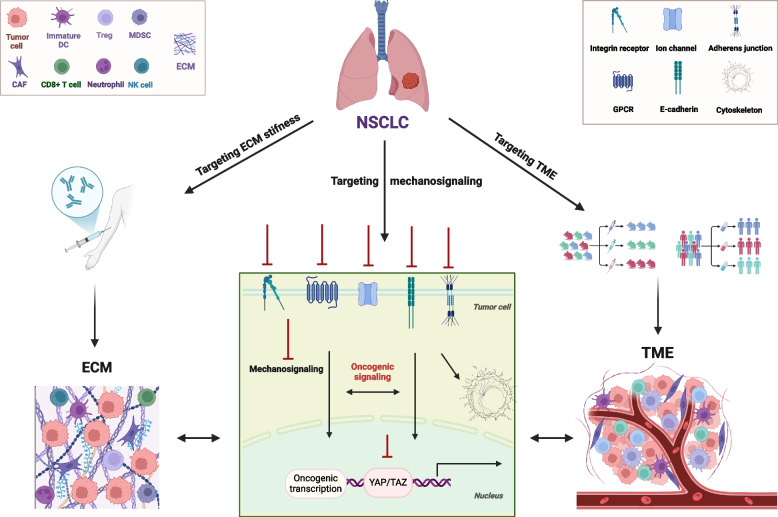
Targeting tumor mechanical properties in NSCLC. Emerging mechanobiological features are crucial for tumor − TME interactions in NSCLC. Modulation of ECM stiffness drives tumor cell properties and unearths candidate targets for treatment. Mechanosignaling-engaged protein molecules in tumor cells and TME cells are potential targets which are being evaluated in preclinical and clinical tests. CAF: cancer-associated fibroblasts; CD8 + T cells: cytotoxic T lymphocytes; ECM: extracellular matrix; GPCR: G protein-coupled receptors; Immature DC: immature dendritic cells; MDSC: myeloid-derived suppressor cells; NK cells: natural-killer cells; NSCLC: non-small cell lung cancer; TAZ: transcriptional coactivator with PDZ-binding motif; TME: tumor microenvironment; Treg: regulatory T cells; YAP: Yes-associated protein. This figure was created based on the tools provided by Biorender (https://biorender.com/)

Overall, it is critical to map the complex signaling circuitries generated from TME, lung cancer cells and mechanical inputs interactions. To do so, mechanobiology research involves multiple disciplines, such as molecular and computational biology, bioinformatics, medicine and bioengineering. Interdisciplinary collaboration is of utmost importance to be able to translate mechanobiological insights into clinical applications. In this vein, coherent funding strategies and the establishment of comprehensive mechanobiology networks are needed to promote exchanges and cooperation among different fields. Elucidating the underpinning mechanisms can be promising for the design of new treatment strategies based on specific tumor profiles and mechanisms of resistance. To this end, these strategies can be categorized as distinct aspects of mechanomedicine, mainly those that target ECM stiffness, mechanosignaling effector molecules and TME (Fig. 1).

## Data Availability

Not applicable.
